# A justification of the invariance of the speed of light by quantum theoretical considerations

**DOI:** 10.1038/s41598-020-80922-w

**Published:** 2021-01-13

**Authors:** Michael Cordin

**Affiliations:** Institute of Textile Chemistry and Textile Physics, University of Innsbruck, 6850 Dornbirn, Austria

**Keywords:** Quantum mechanics, Theoretical physics

## Abstract

Abstract phenomena can often be analysed in a visually clear way by thought experiments. In the present work a new thought experiment is analysed, which describes the emission of a photon by a special mechanism. The analysis leads to the finding, that there is a natural connection between the energy-time uncertainty and the Heisenberg position-momentum uncertainty principle. A deeper analysis of this thought experiment, especially with regard to a justification of the Doppler effect from the uncertainty principle, leads to the conclusion, that the speed of light is invariant. Therefore it seems reasonable to assume, that the origin of relativistic laws lies in non-relativistic quantum mechanics.

## Introduction

The Heisenberg uncertainty principle describes the heart of quantum mechanics, particulary that position and momentum of a particle cannot be measured with infinite accuracy. This is due to the fact, that particles have a wave character. The uncertainty principle was the basis for the development of the Copenhagen interpretation of quantum mechanics^1,2^. The uncertainty of position and momentum can be expressed by the standard deviation $\sigma _{x}$ and $\sigma _{p}$ and satisfy the following equation^3^:

1 $$\begin{aligned} \sigma _{x}\cdot \sigma _{p}\ge \frac{\hbar }{2} \end{aligned}$$

The interpretation of the energy-time uncertainty principle is controversially discussed, because the time cannot be represented without contradiction by a time operator. Therefore the energy-time uncertainty principle is often applied to explain special physical phenomena, for example the appearance of the natural broadening of the emission spectral line of an excited and unstable state. A short lifetime of the excited state is connected with a larger energy uncertainty and therefore with a broader emission spectral line. A closer analysis shows, that this spectral line can be described by a Lorentz curve and its width is inversely proportional to the lifetime of the excited state^4,5^.

## Discussion of the thought experiment

Now imagine a photon, which is reflected forwards and backwards between two mirrors. The right mirror is a little bit light-transmitting and therefore the probability, that the photon is reflected by this mirror is *a*. If the photon is *n* times reflected by this mirror, then the probability of the photon to be still between the two mirrors is:

2 $$\begin{aligned} P(n)=a^{n} \end{aligned}$$

Formula 2 can also be expressed as a function of time, because the number of reflections correlates with time:

3 $$\begin{aligned} P(t)=e^{-A\cdot t} \end{aligned}$$

So the probability to find the photon between the two mirror can be described by an exponential function, as shown in formula 3. For a very small time interval between the reflections the dicretization effect can be neglected and *P*(*t*) can be described by formula 3. So this system can be regarded as a model system similar to an unstable and excited state, which can emit a photon. Now imagine, that the transmittance of the right mirror is increased with time, so that *P*(*t*) satisfies the following equation (for $0\le t \le 1/b$):

4 $$\begin{aligned} P(t)=1-b\cdot t \end{aligned}$$

According to formula 4 the emission rate is constant and after a time $t=1/b$, the photon has been emitted with a probability of 100%. So the emission of the photon occurs during a period of time $\Delta t$ and the emitted photon is delocalized over an area of $\Delta x=c\cdot \Delta t$ along the flight direction. The standard deviation of *x* for the delocalized photon can be easily calculated from the standard deviation of the time, according to the following equation:

5 $$\begin{aligned} \sigma _{x}=c\cdot \sigma _{t} \end{aligned}$$

By substituting this into the formula for the position-momentum uncertainty formula, one obtains the following equation:

6 $$\begin{aligned} c\cdot \sigma _{t}\cdot \sigma _{p}\ge const \end{aligned}$$

A more delocalized photon has a smaller uncertainty with respect to the momentum. The energy of a photon is $E=pc$ and therefore formula 6 can be transformed to:

7 $$\begin{aligned} \sigma _{t}\cdot \sigma _{E}\ge const \end{aligned}$$

The emission time of the photon is uncertain over a period of time $\Delta t$ and consequently the photon is delocalized. The extent of delocalization determines the uncertainty with respect to the momentum along the flight direction, which corresponds to the total momentum of the photon and consequently also influences the energy. So the delocalization (along the flight direction) determines the size of the momentum interval, which determines how many wave-functions have to be added up by superposition to reproduce the given delocalization. For photons the formula $E=pc$ is the result of the relativistic invariant Maxwell equations. But the energy-time uncertainty relation is a generally accepted result of non-relativistic quantum mechanics and the considered model system shows, that the natural broadening of the emission spectral line for photons can only be explained in a consistent way, if the formula $E=pc$ holds true for photons (with *c* as a constant). If the photon energy would not be linear proportional to its momentum, then a wrong relation for the energy-time uncertainty would be the result. So this can be regarded as a first indication, that the speed of light is invariant. The analysis shows also that the energy-time uncertainty and the position-momentum uncertainty relation can be regarded as the two sides of the same coin. The results for this model system can be transferred to the emission of an excited and unstable system. The shorter the lifetime of the excited state, the smaller the delocalization of the photon is and consequently the energy uncertainty is larger. Now imagine an observer, which moves away from the mirrors along the direction of the emitted photon with the velocity *v*. From the point of view of this observer, the emitted photon moves with the invariant speed of light and therefore the emitted photon is delocalized over an area $\Delta x^{\prime }=(c+v)\Delta t$. A more delocalized photon means, that its momentum uncertainty is decreased and consequently (for a photon) also its energy uncertainty in a defined way. For a small velocity *v*, the time dilation of $\Delta t$ can be neglected for the considered thought experiment, because the dilated $\Delta t$ can be described by a Taylor series and the first correction term is proportional to $v^{2}/c^{2}$. The photon is now delocalized over a larger area, compared to the point of view of an observer at rest. The delocalization is increased by an factor $1+v/c$ and therefore the energy uncertainty of this photon is decreased by an factor $1/(1+v/c)$ and satisfies the following equation:

8 $$\begin{aligned} \Delta E^{\prime }=\Delta E\cdot \frac{1}{1+\frac{v}{c}} \end{aligned}$$

The derivation of Eq. (8) assumes the preparation of a photon in a state of fixed uncertainty. The decrease in the energy uncertainty corresponds exactly to the prediction, if the same situation is analysed with the Doppler effect. The Doppler effect decreases the natural broadening of the spectral line of the emitted photon, but the natural broadening is the result of the Heisenberg uncertainty principle and therefore the photon has to be delocalized over a larger area and this is only possible if the speed of light is invariant. Due to the increased delocalization of the photon, its momentum and energy uncertainty decreases exactly to the prediction of the Doppler effect. Now imagine that the emitted and delocalized photon overtakes the moving observer and is then reflected back by a mirror at rest. Form the point of view of the moving observer, the reflected photon is now delocalized over an area $\Delta x^{\prime }=(c-v)\Delta t$ and so its energy uncertainty is increased by a factor of $1/(1-v/c)$, in accordance with the prediction of the Doppler effect.

The present analysis of the thought experiment assumes that the position-momentum uncertainty relation is the same for the unmoving and moving observers, namely $\Delta x\Delta p=\Delta x^{\prime }\Delta p^{\prime }$. This assumption can be justified by the following consideration: Now imagine a system with an observer at rest and a photon (1) with the energy *E*. The photon (1) moves along the direction of the x-axis. Now imagine a second system with a moving observer and photon (2) with the energy *E* (from the point of view of the moving observer). This observer and photon (2) move also along the x-axis. Due to the fact, that the speed of light is invariant, the two systems are equivalent from the point of view of the respective observers. The same uncertainty in momentum for photon (1) and photon (2) corresponds therefore to the same delocalization of the photon (from the point of view of the respective observers). If this would not be the case, an absolute velocity could be determined for the observers and the two systems would not be equivalent. This shows, that the same position-momentum uncertainty relation holds true for the two situations with the unmoving and moving observer.

## Conclusion

According to the present thought experiment, the position-momentum uncertainty relation is more fundamental than the energy-time uncertainty relation, although the two phenomena can be transformed into each other for special physical situations. The extend of delocalization determines the extend of momentum uncertainty and determines the size of the momentum interval, which determines how many wave-functions have to be added up by superposition to reproduce the given delocalization. The analysis shows also, that the extent of delocalization of an emitted photon depends on the point of view of the observer, if the speed of light is regarded as invariant. Under these conditions, the influence of the Doppler effect on the natural broadening of the spectral line of the emitted photon can be explained by the Heisenberg uncertainty principle. So the interpretation of the present thought experiment seems only to be possible without contradiction, if the speed of light is invariant.

## References

[1] Heisenberg W (1927). Über den anschaulichen inhalt der quantentheoretischen kinematik und mechanik. Z. Phys..

[2] Greiner W (2005). Quantenmechanik.

[3] Kennard E (1927). Zur quantenmechanik einfacher bewegungstypen. Z. Phys..

[4] Cohen-Tannoudji C, Dupont-Roc J, Grynberg G (1992). Atom–Photon Interactions—Basic Processes and Applications.

[5] Messiah A (1985). Quantenmechanik 2.

